# Supplementary material for: Pain in Long-Term Cancer Survivors: Prevalence and Impact in a Cohort Composed Mostly of Breast Cancer Survivors
Source: Cancers (Basel). 2024 Apr 20;16(8):1581. doi: 10.3390/cancers16081581 (PMC11049399; doi:10.3390/cancers16081581)
Supplement: Supplementary file 1 [file cancers-16-01581-s001.zip › cancers-2958597-supplementary.pdf]

xTitle page

Title: Pain in Long-Term Cancer Survivors: A Cohort Study of Prevalence and Impact

Authors

Concepción Pérez* [a], Dolores Ochoa* [a], Noelia Sánchez [a], Ana-Isabel Ballesteros [b], Olga Donay [b], Isabel López [a], Rebeca Mondéjar [b], Thiago Carnaval [c], Jesús Villoria [c], Ramón Colomer [b]

[a] Pain Clinic, Hospital de La Princesa, Madrid, Spain

[b] Department of Clinical Oncology, Hospital de la Princesa, Madrid, Spain

[c] Department of Design and Biometrics, Medicxact, S.L., Alpedrete, Spain

* Contributed equally

Short title: Pain Prevalence and Impact in Long-Term Cancer Survivors

Address for correspondence

Concepción Pérez, Pain Clinic, Hospital de La Pricesa, calle de Diego de León 62, 28006, Madrid, Spain, email: concha.phte@gmail.com.

Category of submission: Original article.

Funding sources: This research has received funds from Grünenthal Pharma S.A., who did not have any direct corporate role in the design, analysis, interpretation of results, or preparation of the manuscript.

Conflict of interest disclosures: The authors do not have any conflict of interest to report.

Significance: Pain in long-term cancer survivors is associated to reductions of quality of life as well as emotional and professional performance. The neuropathic component is often underdiagnosed and/or undertreated. Adopting appropriate neuropathic pain diagnostic tools should be standard clinical practice and targeting the neuropathic component seems a good and underused therapeutic approach to improve cancer survivors' health outcomes.

Table of Contents

Title page 1

Table of Contents...3

Supplementary Tables..4

Supplementary Figures 17

Supplementary Tables

Table S1: Correlates of EQ-5D Quality of Life Index (multivariable linear Gaussian regression)

	Estimate [a]	95% confidence interval Lower [a]	Upper [a]	P-value
Intercept	**1.14**	**0.90**	**1.38**	**<0.001**
Having probable neuropathic pain (DN4≥4)	**−0.38**	**−0.43**	**−0.32**	**<0.001**
Having probable nociceptive pain (DN4<4)	**−0.23**	**−0.29**	**−0.17**	**<0.001**
Age, change per additional year	0.00	0.00	0.00	0.705
Male gender	0.02	−0.07	0.11	0.654
BMI, change per 1-unit (kg/m^2) increase	**−0.01**	**−0.01**	**0.00**	**0.002**
Being married	0.00	−0.04	0.05	0.875
Having paid job	0.06	0.00	0.11	0.063
Having superior studies	0.01	−0.04	0.05	0.802
Smoking or routine alcohol intake	−0.02	−0.07	0.03	0.418
Having any chronic disease	−0.04	−0.09	0.00	0.056
Time since cancer onset, chg. per year	0.00	−0.01	0.00	0.656
Having had breast cancer	−0.02	−0.12	0.07	0.625
Having had advanced cancer	−0.01	−0.07	0.05	0.669
Two surgeries (vs. one or none)	0.02	−0.03	0.06	0.534
Three or more surgeries (vs. one or none)	−0.05	−0.12	0.02	0.171
One or two chemotherapies (vs. none)	0.00	−0.08	0.09	0.951
Three or more chemotherapies (vs. none)	−0.02	−0.12	0.09	0.777
Radiotherapy	**−0.06**	**−0.11**	**−0.01**	**0.025**
Vinca alkaloids or platinum compounds	0.00	−0.09	0.09	0.986
Taxanes	−0.01	−0.07	0.04	0.629
Anthracyclines	0.03	−0.07	0.14	0.548
Nitrogen mustards	0.03	−0.08	0.14	0.625
Aromatase inhibitors	**0.07**	**0.02**	**0.12**	**0.008**

Abbreviations: BMI: body mass index, chg.: change, kg: kilogram, m: metre.

[a] An identity link was used in this binary model; thus, the estimates are in the response scale.

Table S2: Correlates of EQ-5D Health Status score (multivariable linear Gaussian regression)

	Estimate [a]	95% confidence interval		P-value
		Lower [a]	Upper [a]	
Intercept	**89.44**	**68.59**	**110.29**	**<0.001**
Having probable neuropathic pain (DN4≥4)	**−14.79**	**−19.45**	**−10.14**	**<0.001**
Having probable nociceptive pain (DN4<4)	**−7.71**	**−12.74**	**−2.68**	**0.003**
Age, change per additional year	−0.12	−0.34	0.10	0.303
Male gender	4.78	−3.03	12.59	0.230
BMI, change per 1-unit (kg/m^2) increase	−0.20	−0.60	0.21	0.341
Being married	2.13	−1.58	5.84	0.261
Having paid job	2.65	−2.38	7.69	0.302
Having superior studies	0.12	−3.93	4.16	0.955
Smoking or routine alcohol intake	−1.01	−5.31	3.30	0.647
Having any chronic disease	**−6.96**	**−10.88**	**−3.03**	**0.001**
Time since cancer onset, chg. per year	−0.02	−0.38	0.34	0.921
Having had breast cancer	−1.14	−9.35	7.06	0.785
Having had advanced cancer	0.83	−4.24	5.89	0.750
Two surgeries (vs. one or none)	4.15	−0.06	8.36	0.054
Three or more surgeries (vs. one or none)	4.41	−1.65	10.47	0.154
One or two chemotherapies (vs. none)	4.09	−3.36	11.53	0.282
Three or more chemotherapies (vs. none)	1.73	−7.52	10.97	0.715
Radiotherapy	−4.25	−8.65	0.16	0.059
Vinca alkaloids or platinum compounds	−1.51	−8.93	5.91	0.689
Taxanes	−0.06	−4.58	4.47	0.979
Anthracyclines	0.67	−8.44	9.77	0.886
Nitrogen mustards	3.90	−5.55	13.35	0.419
Aromatase inhibitors	2.15	−2.36	6.65	0.350

Abbreviations: BMI: body mass index, chg.: change, kg: kilogram, m: metre.

[a] An identity link was used in this binary model; thus, the estimates are in the response scale.

Table S3: Correlates of HADS anxiety score (multivariable linear Gaussian regression)

	Estimate [a]	95% confidence interval Lower [a]	Upper [a]	P-value
Intercept	**10.17**	**4.27**	**16.07**	**0.001**
Having probable neuropathic pain (DN4≥4)	**3.89**	**2.56**	**5.22**	**<0.001**
Having probable nociceptive pain (DN4<4)	**1.92**	**0.50**	**3.33**	**0.008**
Age, change per additional year	**−0.07**	**−0.13**	**−0.01**	**0.024**
Male gender	0.70	−1.51	2.92	0.535
BMI, change per 1-unit (kg/m^2) increase	−0.02	−0.13	0.09	0.714
Being married	0.06	−0.99	1.11	0.906
Having paid job	−0.64	−2.06	0.77	0.374
Having superior studies	−0.71	−1.85	0.43	0.223
Smoking or routine alcohol intake	0.24	−0.98	1.46	0.698
Having any chronic disease	−0.23	−1.35	0.89	0.687
Time since cancer onset, chg. per year	**0.10**	**0.00**	**0.20**	**0.049**
Having had breast cancer	0.12	−2.22	2.46	0.922
Having had advanced cancer	0.45	−0.99	1.89	0.538
Two surgeries (vs. one or none)	**−1.49**	**−2.67**	**−0.31**	**0.014**
Three or more surgeries (vs. one or none)	−1.60	−3.33	0.12	0.069
One or two chemotherapies (vs. none)	−1.91	−4.02	0.21	0.077
Three or more chemotherapies (vs. none)	−1.86	−4.48	0.75	0.163
Radiotherapy	−0.81	−2.05	0.43	0.202
Vinca alkaloids or platinum compounds	−1.00	−3.11	1.10	0.351
Taxanes	0.20	−1.08	1.48	0.758
Anthracyclines	−0.98	−3.57	1.62	0.460
Nitrogen mustards	2.63	−0.08	5.33	0.057
Aromatase inhibitors	−0.67	−1.93	0.59	0.300

Abbreviations: BMI: body mass index, chg.: change, kg: kilogram, m: metre.

[a] An identity link was used in this binary model; thus, the estimates are in the response scale.

Table S4: Correlates of HADS depression score (multivariable linear Gaussian regression)

	Estimate [a]	95% confidence interval Lower [a]	Upper [a]	P-value
Intercept	−0.52	−5.72	4.68	0.844
Having probable neuropathic pain (DN4≥4)	**3.13**	**1.95**	**4.30**	**<0.001**
Having probable nociceptive pain (DN4<4)	**1.76**	**0.51**	**3.00**	**0.006**
Age, change per additional year	0.02	−0.04	0.07	0.575
Male gender	0.54	−1.44	2.52	0.593
BMI, change per 1-unit (kg/m^2) increase	**0.12**	**0.02**	**0.21**	**0.023**
Being married	**−1.02**	**−1.95**	**−0.10**	**0.030**
Having paid job	−1.04	−2.29	0.21	0.103
Having superior studies	−0.71	−1.72	0.30	0.168
Smoking or routine alcohol intake	0.41	−0.67	1.49	0.456
Having any chronic disease	0.68	−0.31	1.67	0.180
Time since cancer onset, chg. per year	0.07	−0.02	0.16	0.133
Having had breast cancer	−0.10	−2.17	1.97	0.926
Having had advanced cancer	0.48	−0.79	1.75	0.455
Two surgeries (vs. one or none)	−0.62	−1.66	0.43	0.247
Three or more surgeries (vs. one or none)	−0.39	−1.91	1.13	0.612
One or two chemotherapies (vs. none)	−1.07	−2.93	0.79	0.261
Three or more chemotherapies (vs. none)	−1.34	−3.65	0.97	0.255
Radiotherapy	−0.21	−1.30	0.89	0.714
Vinca alkaloids or platinum compounds	−0.47	−2.33	1.39	0.623
Taxanes	0.95	−0.18	2.08	0.099
Anthracyclines	−0.83	−3.11	1.46	0.478
Nitrogen mustards	1.41	−0.98	3.79	0.248
Aromatase inhibitors	**−1.23**	**−2.34**	**−0.12**	**0.030**

Abbreviations: BMI: body mass index, chg.: change, kg: kilogram, m: metre.

[a] An identity link was used in this binary model; thus, the estimates are in the response scale.

Table S5: Correlates of HADS total score (multivariable linear Gaussian regression)

	Estimate [a]	95% confidence interval		P-value
		Lower [a]	Upper [a]	
Intercept	9.66	−0.24	19.55	0.056
Having probable neuropathic pain (DN4≥4)	**7.01**	**4.78**	**9.24**	**<0.001**
Having probable nociceptive pain (DN4<4)	**3.69**	**1.32**	**6.06**	**0.002**
Age, change per additional year	−0.06	−0.16	0.05	0.279
Male gender	0.97	−2.79	4.74	0.611
BMI, change per 1-unit (kg/m^2) increase	0.10	−0.09	0.29	0.308
Being married	−1.01	−2.77	0.76	0.264
Having paid job	−1.68	−4.05	0.70	0.167
Having superior studies	−1.34	−3.26	0.58	0.172
Smoking or routine alcohol intake	0.69	−1.36	2.74	0.510
Having any chronic disease	0.38	−1.50	2.26	0.691
Time since cancer onset, chg. per year	**0.17**	**0.01**	**0.34**	**0.043**
Having had breast cancer	−0.11	−4.04	3.83	0.958
Having had advanced cancer	0.96	−1.45	3.38	0.435
Two surgeries (vs. one or none)	**−2.08**	**−4.06**	**−0.09**	**0.040**
Three or more surgeries (vs. one or none)	−1.96	−4.85	0.94	0.185
One or two chemotherapies (vs. none)	−2.98	−6.52	0.56	0.099
Three or more chemotherapies (vs. none)	−3.28	−7.67	1.11	0.143
Radiotherapy	−0.95	−3.04	1.13	0.370
Vinca alkaloids or platinum compounds	−1.54	−5.08	2.00	0.394
Taxanes	1.19	−0.96	3.34	0.277
Anthracyclines	−1.80	−6.15	2.56	0.418
Nitrogen mustards	4.02	−0.52	8.56	0.083
Aromatase inhibitors	−1.87	−3.99	0.24	0.083

Abbreviations: BMI: body mass index, chg.: change, kg: kilogram, m: metre.

[a] An identity link was used in this binary model; thus, the estimates are in the response scale.

Table S6: Correlates of PCS total score (multivariable linear Gaussian regression)

	Estimate [a]	95% confidence interval Lower [a]	Upper [a]	P-value
Intercept	3.59	−8.44	15.63	0.558
Having probable neuropathic pain (DN4≥4)	**13.07**	**10.32**	**15.81**	**<0.001**
Having probable nociceptive pain (DN4<4)	**5.18**	**2.30**	**8.07**	**<0.001**
Age, change per additional year	−0.05	−0.18	0.07	0.422
Male gender	2.78	−1.85	7.41	0.239
BMI, change per 1-unit (kg/m^2) increase	0.08	−0.15	0.31	0.503
Being married	−0.79	−2.95	1.38	0.476
Having paid job	−1.72	−4.63	1.18	0.245
Having superior studies	−2.15	−4.50	0.21	0.074
Smoking or routine alcohol intake	−0.47	−2.95	2.00	0.708
Having any chronic disease	**2.76**	**0.46**	**5.07**	**0.019**
Time since cancer onset, chg. per year	−0.05	−0.26	0.16	0.621
Having had breast cancer	3.17	−1.68	8.02	0.200
Having had advanced cancer	1.01	−1.93	3.95	0.499
Two surgeries (vs. one or none)	**−2.90**	**−5.34**	**−0.47**	**0.019**
Three or more surgeries (vs. one or none)	**−3.84**	**−7.40**	**−0.29**	**0.034**
One or two chemotherapies (vs. none)	0.42	−3.93	4.76	0.851
Three or more chemotherapies (vs. none)	2.59	−2.77	7.96	0.343
Radiotherapy	−0.67	−3.21	1.87	0.604
Vinca alkaloids or platinum compounds	−0.78	−5.14	3.59	0.728
Taxanes	0.16	−2.44	2.77	0.903
Anthracyclines	0.37	−4.90	5.65	0.889
Nitrogen mustards	−0.07	−5.56	5.42	0.979
Aromatase inhibitors	**−3.62**	**−6.21**	**−1.04**	**0.006**

Abbreviations: BMI: body mass index, chg.: change, kg: kilogram, m: metre.

[a] An identity link was used in this binary model; thus, the estimates are in the response scale.

Table S7: Correlates of WPAI-GH paid employment (multivariable logistic regression)

		95% confidence interval		
	Estimate [a]	Lower [a]	Upper [a]	P-value
Intercept	**14.67**	**8.55**	**20.80**	**<0.001**
Having probable neuropathic pain (DN4≥4)	**−1.47**	**−2.66**	**−0.27**	**0.016**
Having probable nociceptive pain (DN4<4)	0.13	−1.10	1.36	0.839
Age, change per additional year	**−0.25**	**−0.33**	**−0.17**	**<0.001**
Male gender	2.47	−0.41	5.35	0.093
BMI, change per 1-unit (kg/m^2) increase	−0.02	−0.12	0.08	0.746
Being married	**−0.94**	**−1.84**	**−0.03**	**0.043**
Having paid job	−0.04	−1.09	1.01	0.947
Having superior studies	0.06	−0.89	1.00	0.907
Smoking or routine alcohol intake	−0.15	−1.10	0.80	0.756
Having any chronic disease	−0.04	−0.14	0.06	0.429
Time since cancer onset, chg. per year	0.77	−2.20	3.73	0.612
Having had breast cancer	−0.05	−1.25	1.15	0.933
Having had advanced cancer	0.97	−0.07	2.01	0.068
Two surgeries (vs. one or none)	1.08	−0.51	2.67	0.183
Three or more surgeries (vs. one or none)	0.22	−1.67	2.11	0.821
One or two chemotherapies (vs. none)	−0.12	−2.41	2.17	0.918
Three or more chemotherapies (vs. none)	−0.40	−1.51	0.72	0.484
Radiotherapy	−0.80	−3.06	1.47	0.491
Vinca alkaloids or platinum compounds	−0.14	−1.22	0.94	0.797
Taxanes	−1.74	−3.88	0.40	0.110
Anthracyclines	**2.24**	**0.12**	**4.35**	**0.039**
Nitrogen mustards	−0.75	−1.79	0.30	0.163
Aromatase inhibitors	**14.67**	**8.55**	**20.80**	**<0.001**

Abbreviations: BMI: body mass index, chg.: change, kg: kilogram, m: metre.

[a] A logit link was used in this binary model; thus, the estimates are log odds ratios.

Table S8: Correlates of WPAI-GH presenteeism score (multivariable linear Gaussian regression)

	Estimate [a]	95% confidence interval Lower [a]	Upper [a]	P-value
Intercept	**85.70**	**29.26**	**142.15**	**0.003**
Having probable neuropathic pain (DN4≥4)	1.16	−11.11	13.42	0.853
Having probable nociceptive pain (DN4<4)	3.66	−9.42	16.74	0.584
Age, change per additional year	**−1.03**	**−1.76**	**−0.29**	**0.006**
Male gender	−29.57	−62.02	2.87	0.074
BMI, change per 1-unit (kg/m^2) increase	0.68	−0.43	1.78	0.232
Being married	**−10.12**	**−19.52**	**−0.73**	**0.035**
Having paid job	**−14.48**	**−27.42**	**−1.54**	**0.028**
Having superior studies	−7.16	−17.67	3.35	0.182
Smoking or routine alcohol intake	**−9.76**	**−18.64**	**−0.88**	**0.031**
Having any chronic disease	0.38	−0.90	1.65	0.562
Time since cancer onset, chg. per year	−29.39	−63.00	4.22	0.087
Having had breast cancer	2.04	−10.42	14.51	0.748
Having had advanced cancer	−1.68	−13.64	10.28	0.783
Two surgeries (vs. one or none)	7.35	−8.66	23.37	0.368
Three or more surgeries (vs. one or none)	11.12	−12.65	34.90	0.359
One or two chemotherapies (vs. none)	27.29	−1.64	56.21	0.064
Three or more chemotherapies (vs. none)	8.55	−5.24	22.34	0.224
Radiotherapy	**−30.58**	**−57.76**	**−3.40**	**0.027**
Vinca alkaloids or platinum compounds	−5.49	−19.10	8.11	0.429
Taxanes	−15.26	−34.25	3.73	0.115
Anthracyclines	0.66	−22.43	23.75	0.955
Nitrogen mustards	−11.09	−22.80	0.61	0.063
Aromatase inhibitors	**85.70**	**29.26**	**142.15**	**0.003**

Abbreviations: BMI: body mass index, chg.: change, kg: kilogram, m: metre.

[a] An identity link was used in this binary model; thus, the estimates are in the response scale.

Table S9: Correlates of WPAI-GH disability score (multivariable linear Gaussian regression)

		95% confidence interval		
	Estimate [a]	Lower [a]	Upper [a]	P-value
Intercept	**−43.31**	**−72.24**	**−14.38**	**0.003**
Having probable neuropathic pain (DN4≥4)	6.70	−0.57	13.97	0.071
Having probable nociceptive pain (DN4<4)	**10.41**	**2.63**	**18.20**	**0.009**
Age, change per additional year	**0.43**	**0.13**	**0.73**	**0.004**
Male gender	−0.60	−12.77	11.56	0.923
BMI, change per 1-unit (kg/m^2) increase	**1.00**	**0.38**	**1.63**	**0.002**
Being married	−1.58	−7.31	4.14	0.588
Having paid job	0.25	−6.04	6.53	0.939
Having superior studies	−2.17	−8.90	4.57	0.528
Smoking or routine alcohol intake	0.50	−5.68	6.67	0.875
Having any chronic disease	−0.35	−0.90	0.20	0.210
Time since cancer onset, chg. per year	1.98	−10.86	14.83	0.762
Having had breast cancer	6.28	−1.67	14.23	0.121
Having had advanced cancer	−4.15	−10.66	2.37	0.212
Two surgeries (vs. one or none)	−6.40	−15.91	3.11	0.187
Three or more surgeries (vs. one or none)	**16.78**	**5.12**	**28.44**	**0.005**
One or two chemotherapies (vs. none)	**17.92**	**3.49**	**32.35**	**0.015**
Three or more chemotherapies (vs. none)	0.40	−6.45	7.25	0.909
Radiotherapy	−0.60	−12.24	11.03	0.919
Vinca alkaloids or platinum compounds	−2.57	−9.61	4.47	0.474
Taxanes	3.44	−10.83	17.72	0.636
Anthracyclines	−12.97	−27.84	1.90	0.087
Nitrogen mustards	2.27	−4.59	9.13	0.517
Aromatase inhibitors	**−43.31**	**−72.24**	**−14.38**	**0.003**

Abbreviations: BMI: body mass index, chg.: change, kg: kilogram, m: metre.

[a] An identity link was used in this binary model; thus, the estimates are in the response scale.

Table S10: Correlates of WPAI-Pain paid employment (multivariable logistic regression)

	Estimate [a]	95% confidence interval		P-value
		Lower [a]	Upper [a]	
Intercept	**11.47**	**6.20**	**16.73**	**<0.001**
Having probable neuropathic pain (DN4≥4)	−0.85	−1.89	0.20	0.112
Having probable nociceptive pain (DN4<4)	−0.29	−1.45	0.87	0.623
Age, change per additional year	**−0.21**	**−0.27**	**−0.14**	**<0.001**
Male gender	**2.74**	**0.03**	**5.45**	**0.048**
BMI, change per 1-unit (kg/m^2) increase	−0.04	−0.13	0.05	0.411
Being married	**−1.07**	**−1.91**	**−0.22**	**0.013**
Having paid job	0.13	−0.83	1.09	0.794
Having superior studies	−0.18	−1.05	0.69	0.689
Smoking or routine alcohol intake	−0.13	−1.01	0.75	0.775
Having any chronic disease	−0.05	−0.14	0.05	0.319
Time since cancer onset, chg. per year	2.49	−0.24	5.23	0.074
Having had breast cancer	0.11	−0.97	1.19	0.846
Having had advanced cancer	0.59	−0.34	1.52	0.212
Two surgeries (vs. one or none)	0.35	−1.07	1.77	0.628
Three or more surgeries (vs. one or none)	1.01	−0.73	2.75	0.256
One or two chemotherapies (vs. none)	0.54	−1.57	2.64	0.616
Three or more chemotherapies (vs. none)	−0.26	−1.29	0.77	0.618
Radiotherapy	0.12	−1.92	2.16	0.905
Vinca alkaloids or platinum compounds	−0.58	−1.57	0.41	0.254
Taxanes	−1.50	−3.54	0.54	0.150
Anthracyclines	1.53	−0.44	3.49	0.128
Nitrogen mustards	−0.60	−1.54	0.34	0.210
Aromatase inhibitors	**11.47**	**6.20**	**16.73**	**<0.001**

Abbreviations: BMI: body mass index, chg.: change, kg: kilogram, m: metre.

[a] A logit link was used in this binary model; thus, the estimates are log odds ratios.

Table S11: Correlates of WPAI-Pain disability score (multivariable linear Gaussian regression)

		95% confidence interval		
	Estimate [a]	Lower [a]	Upper [a]	P-value
Intercept	−26.39	−53.19	0.40	0.054
Having probable neuropathic pain (DN4≥4)	**46.06**	**39.36**	**52.76**	**<0.001**
Having probable nociceptive pain (DN4<4)	**31.40**	**24.23**	**38.58**	**<0.001**
Age, change per additional year	0.16	−0.11	0.43	0.255
Male gender	−9.09	−20.40	2.22	0.115
BMI, change per 1-unit (kg/m^2) increase	0.40	−0.17	0.97	0.171
Being married	1.47	−3.80	6.74	0.585
Having paid job	−1.64	−7.44	4.16	0.580
Having superior studies	1.76	−4.45	7.96	0.579
Smoking or routine alcohol intake	1.95	−3.75	7.65	0.503
Having any chronic disease	0.28	−0.23	0.79	0.277
Time since cancer onset, chg. per year	0.93	−10.90	12.77	0.877
Having had breast cancer	3.96	−3.41	11.33	0.293
Having had advanced cancer	−3.99	−9.99	2.01	0.192
Two surgeries (vs. one or none)	−5.43	−14.20	3.34	0.225
Three or more surgeries (vs. one or none)	5.97	−4.78	16.71	0.276
One or two chemotherapies (vs. none)	9.44	−3.85	22.73	0.164
Three or more chemotherapies (vs. none)	1.97	−4.36	8.30	0.542
Radiotherapy	−0.17	−10.92	10.58	0.975
Vinca alkaloids or platinum compounds	3.46	−3.05	9.97	0.297
Taxanes	−5.67	−18.83	7.48	0.398
Anthracyclines	−1.97	−15.67	11.73	0.778
Nitrogen mustards	−4.45	−10.77	1.87	0.168
Aromatase inhibitors	−26.39	−53.19	0.40	0.054

Abbreviations: BMI: body mass index, chg.: change, kg: kilogram, m: metre.

[a] An identity link was used in this binary model; thus, the estimates are in the response scale.

Table S12: Correlates of Insomnia (multivariable logistic regression)

	Estimate [a]	95% confidence interval Lower [a]	Upper [a]	P-value
Intercept	−0.05	−3.24	3.15	0.976
Having probable neuropathic pain (DN4≥4)	**1.44**	**0.70**	**2.17**	**<0.001**
Having probable nociceptive pain (DN4<4)	**0.82**	**0.07**	**1.56**	**0.032**
Age, change per additional year	−0.02	−0.05	0.02	0.303
Male gender	0.07	−1.17	1.31	0.912
BMI, change per 1-unit (kg/m^2) increase	−0.02	−0.09	0.04	0.450
Being married	0.34	−0.23	0.91	0.239
Having paid job	0.22	−0.55	1.00	0.576
Having superior studies	−0.05	−0.67	0.57	0.875
Smoking or routine alcohol intake	0.02	−0.64	0.68	0.945
Having any chronic disease	0.35	−0.26	0.95	0.263
Time since cancer onset, chg. per year	0.04	−0.02	0.10	0.156
Having had breast cancer	0.30	−0.97	1.58	0.641
Having had advanced cancer	−0.25	−1.05	0.56	0.550
Two surgeries (vs. one or none)	−0.40	−1.04	0.25	0.229
Three or more surgeries (vs. one or none)	−0.36	−1.33	0.61	0.463
One or two chemotherapies (vs. none)	0.93	−0.22	2.08	0.112
Three or more chemotherapies (vs. none)	0.96	−0.47	2.39	0.190
Radiotherapy	−0.09	−0.77	0.58	0.784
Vinca alkaloids or platinum compounds	−0.80	−1.96	0.35	0.174
Taxanes	0.04	−0.67	0.75	0.919
Anthracyclines	−0.58	−2.00	0.84	0.422
Nitrogen mustards	−0.03	−1.52	1.46	0.971
Aromatase inhibitors	0.35	−0.34	1.04	0.318

Abbreviations: BMI: body mass index, chg.: change, kg: kilogram, m: metre.

[a] A logit link was used in this binary model; thus, the estimates are log odds ratios.

Table S13: Correlates of Fatigue (multivariable logistic regression)

	Estimate [a]	95% confidence interval Lower [a]	Upper [a]	P-value
Intercept	−1.85	−5.13	1.43	0.269
Having probable neuropathic pain (DN4≥4)	**1.05**	**0.32**	**1.79**	**0.005**
Having probable nociceptive pain (DN4<4)	0.36	−0.41	1.12	0.359
Age, change per additional year	0.00	−0.03	0.04	0.845
Male gender	−0.64	−1.85	0.56	0.297
BMI, change per 1-unit (kg/m^2) increase	0.04	−0.02	0.10	0.210
Being married	−0.41	−0.99	0.17	0.163
Having paid job	0.28	−0.50	1.06	0.489
Having superior studies	0.35	−0.27	0.98	0.269
Smoking or routine alcohol intake	−0.08	−0.76	0.60	0.810
Having any chronic disease	**0.64**	**0.02**	**1.25**	**0.042**
Time since cancer onset, chg. per year	−0.04	−0.10	0.02	0.202
Having had breast cancer	−0.73	−2.04	0.59	0.280
Having had advanced cancer	−0.28	−1.06	0.50	0.485
Two surgeries (vs. one or none)	−0.25	−0.90	0.41	0.461
Three or more surgeries (vs. one or none)	0.11	−0.85	1.07	0.818
One or two chemotherapies (vs. none)	0.78	−0.41	1.96	0.200
Three or more chemotherapies (vs. none)	**1.54**	**0.07**	**3.00**	**0.040**
Radiotherapy	0.62	−0.08	1.32	0.083
Vinca alkaloids or platinum compounds	−0.53	−1.70	0.64	0.372
Taxanes	−0.06	−0.76	0.64	0.861
Anthracyclines	**−1.81**	**−3.27**	**−0.35**	**0.015**
Nitrogen mustards	0.43	−1.08	1.94	0.574
Aromatase inhibitors	0.40	−0.29	1.08	0.257

Abbreviations: BMI: body mass index, chg.: change, kg: kilogram, m: metre.

[a] A logit link was used in this binary model; thus, the estimates are log odds ratios.

Supplementary Figures

Figure S1: Frequencies of common chemotherapeutic agents by study cohort

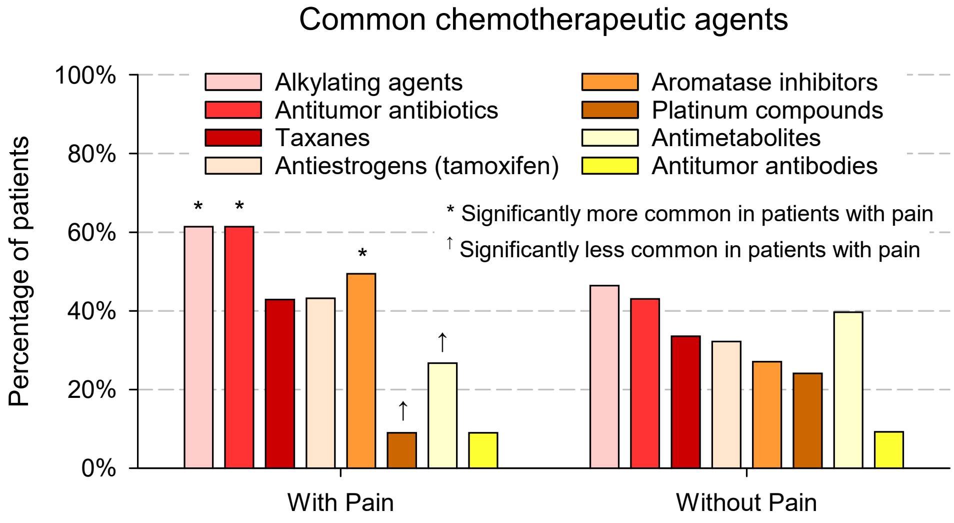

Figure S2: Brief Pain Inventory (BPI) scores' distribution by intensity category

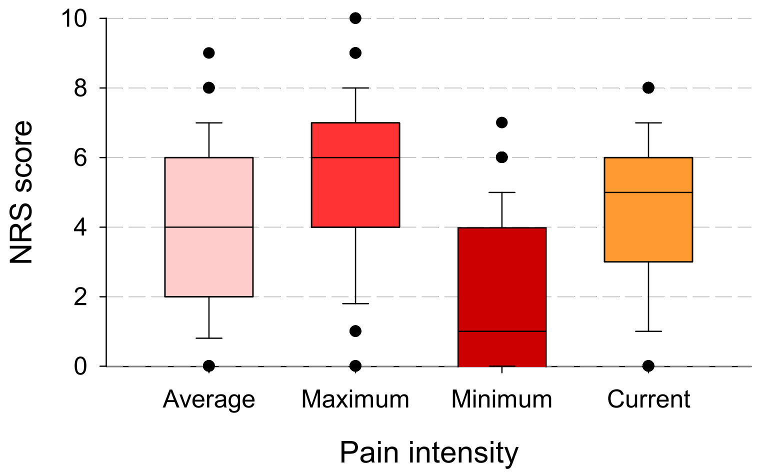

BPI: Brief Pain Inventory. Maximum, Minimum, Average, and "Right Now" intensities refer, respectively, to BPI's questions 3, 4, 5, and 6.

Figure S3: Frequencies of pain locations by pain type

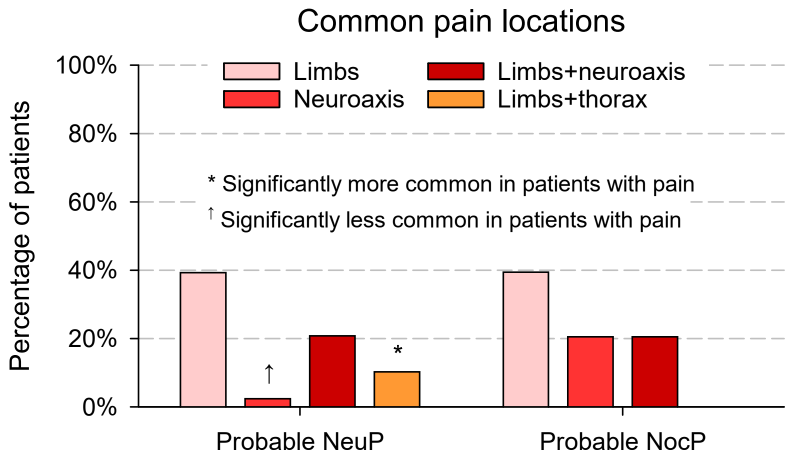

Figure S4: EuroQoL 5 Dimensions

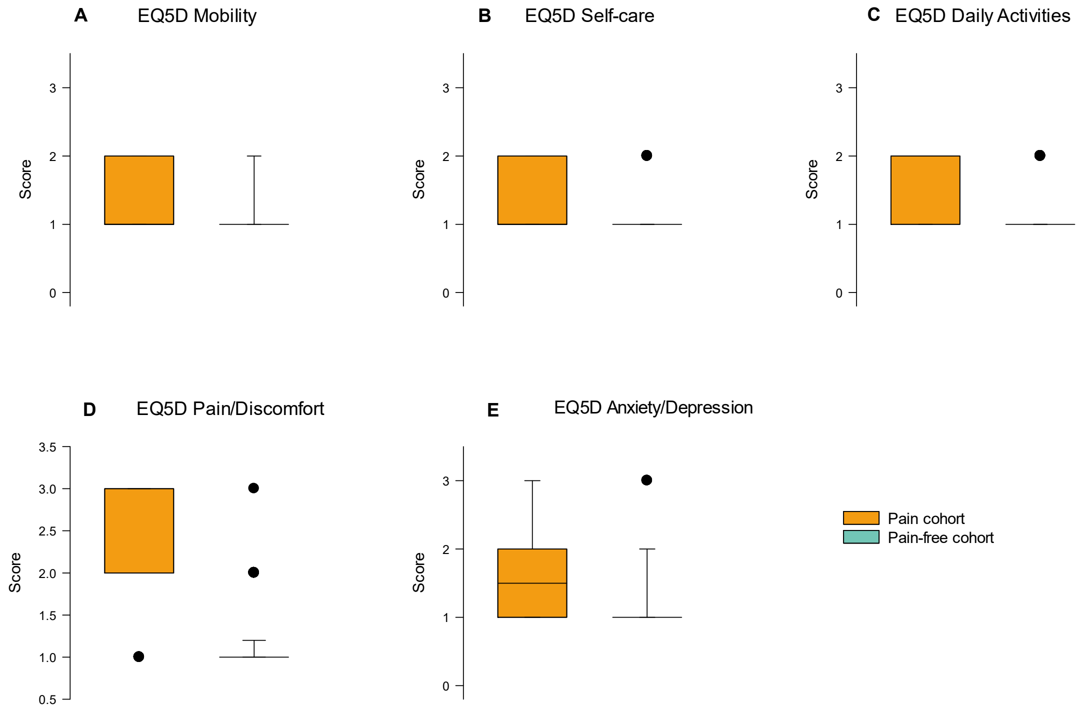

EuroQoL 5 Dimensions instrument. A) Mobility dimension; B) Self-care dimension; C) Daily activities dimension; D) Pain/Discomfort dimension; E) Anxiety/Depression dimension.

Figure S5: EQ5D Health Status Visual Analogue Scale and EQ5D Pain/Discomfort Domain

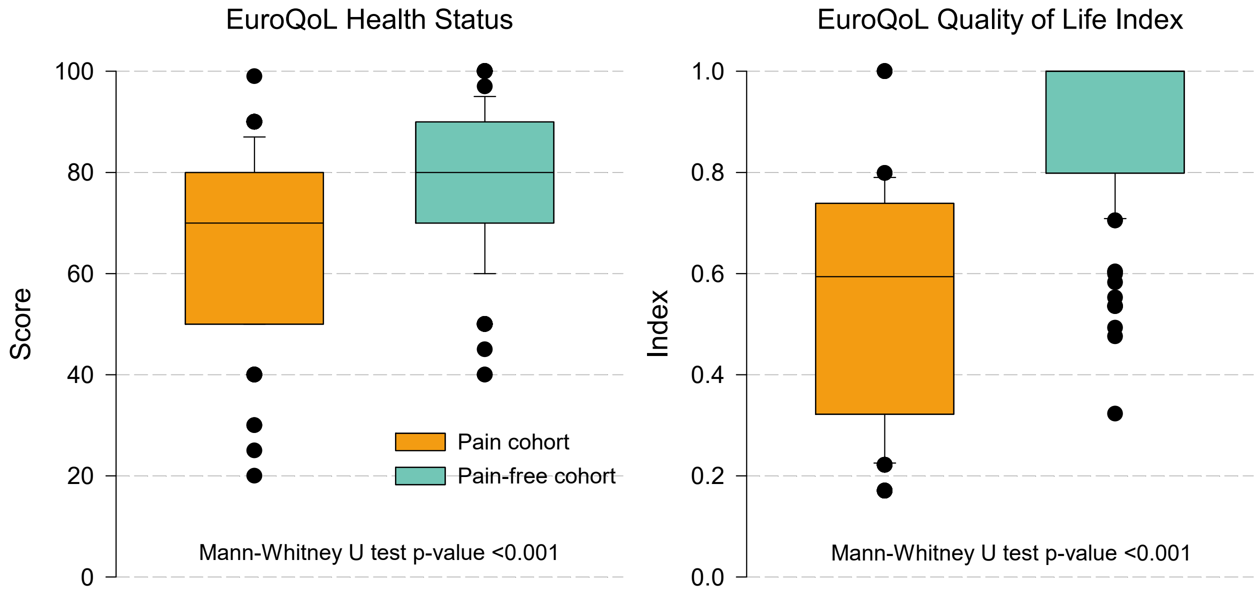

EuroQoL 5 Dimensions instrument. Health Status is scored from zero (worst health status possible) to 100 (best health status possible) and consists of a visual analogue scale. The Quality-of-Life Index is based on individual preferences and the index of preference values for each health state are obtained from general population or patient groups' studies. The Quality-of-Life Index oscillates from one (best health state) to zero (death), although there are negative values that correspond to those health states valued as worse than death.

Figure S6: Number of Patients per category in each HADS subscale

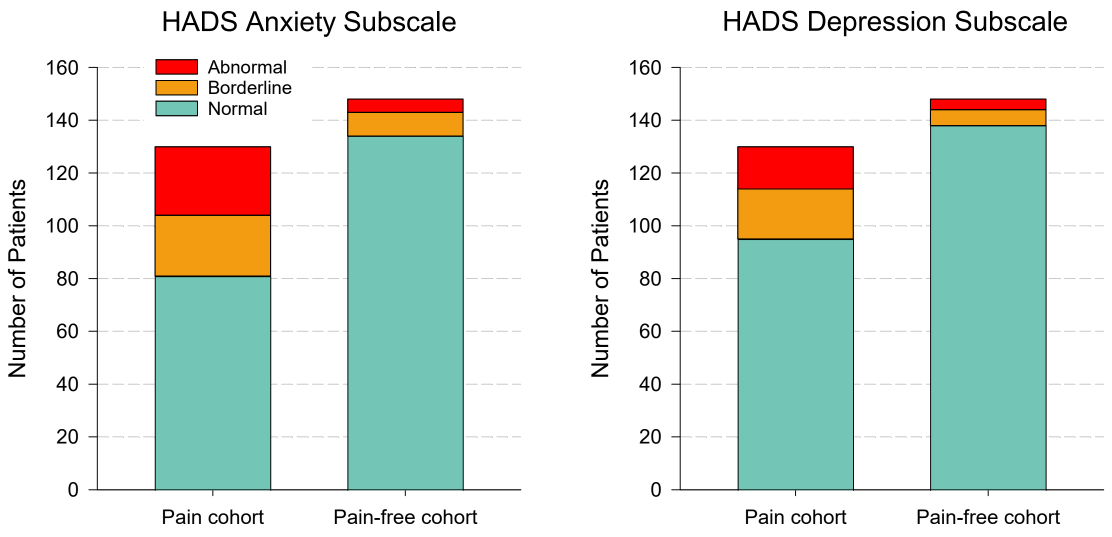

HADS: Hospital Anxiety and Depression Scale. Scores from 0 – 7 are considered normal; scores from 8 – 10 are considered borderline; scores from 11 – 21 are considered abnormal.

Figure S7: Hospital Anxiety and Depression Scale (HADS) Scores

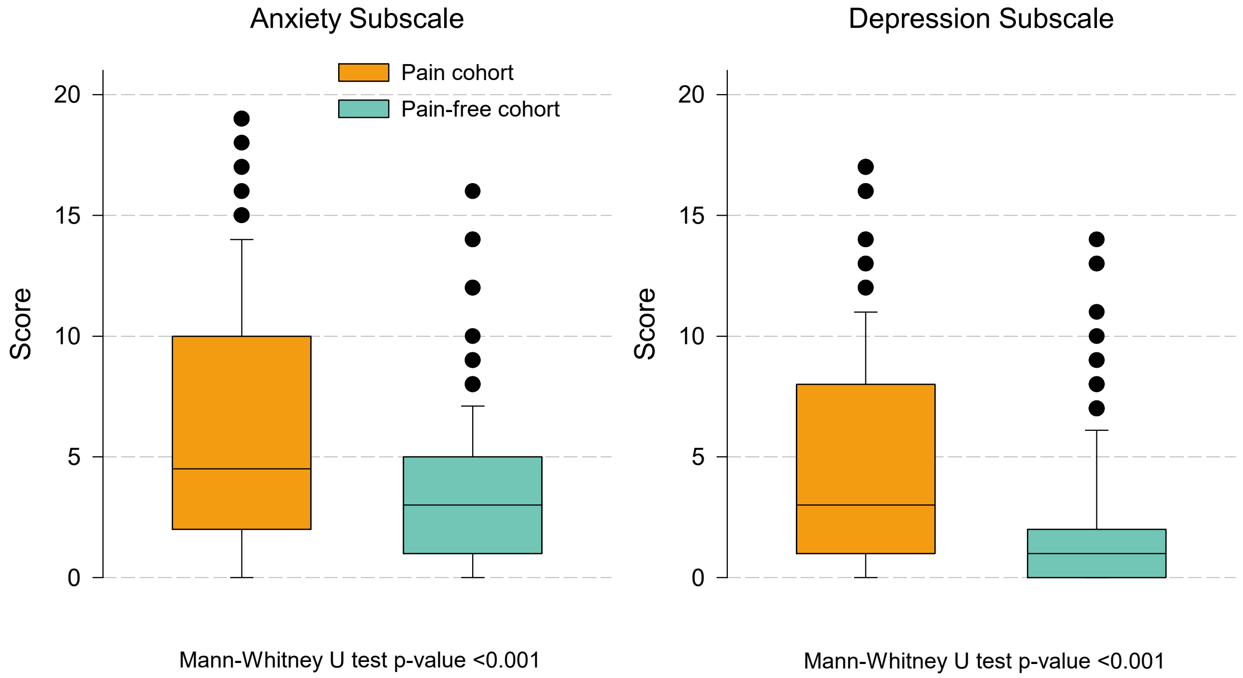

HADS: Hospital Anxiety and Depression Scale.

Figure S8: Components and total PCS scores

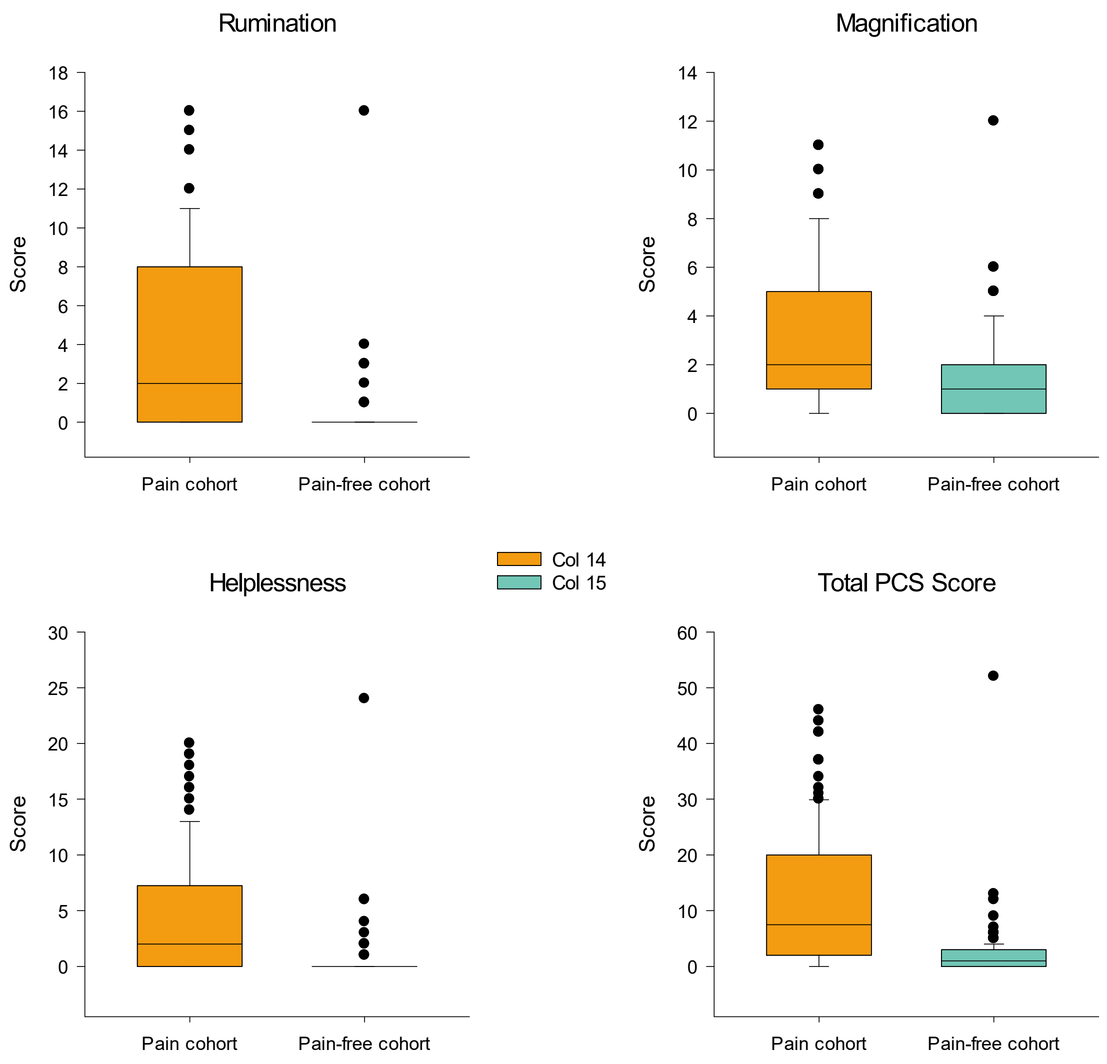

PCS: Pain Catastrophising Scale

Figure S9: Absenteeism and Disability measured with the Work Productivity and Activity Impairment (WPAI) Questionnaire

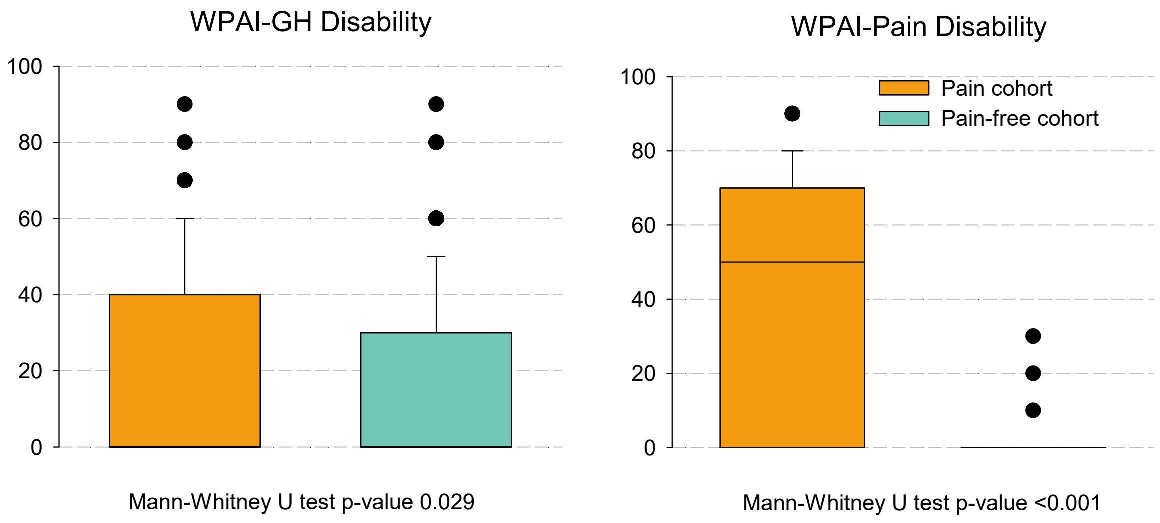

WPAI – GH: Work Productivity and Activity Impairment (WPAI) General Health Questionnaire; WPAI - Pain: WPAI Pain Questionnaire.
